# Early‐life environmental enrichment reduces anxiety and induces sex‐dependent neuroplasticity in hypothalamic oxytocin and vasopressin neurons

**DOI:** 10.1111/jne.70263

**Published:** 2026-09-09

**Authors:** Samantha Costa Amorim Muniz, João Victor Silva Nani, Naima Jamile dos Santos Marciano, Raquel do Nascimento de Souza, Roberto Laureano‐Melo, Wellington da Silva Cortes, Fábio Fagundes da Rocha, André Souza Mecawi

**Affiliations:** ^1^ Department of Physiological Sciences Instituto de Ciências Biológicas e da Saúde, Universidade Federal Rural do Rio de Janeiro Seropédica Brazil; ^2^ Postgraduate Multicentre Program in Physiological Sciences, Instituto de Ciências Biológicas e da Saúde Universidade Federal Rural do Rio de Janeiro Seropédica Brazil; ^3^ Laboratory of Molecular Neuroendocrinology, Department of Biophysics, Escola Paulista de Medicina Universidade Federal de São Paulo São Paulo Brazil

**Keywords:** behaviour, environmental enrichment, neurodevelopment, oxytocin, vasopressin

## Abstract

Early‐life experiences critically shape neuroendocrine systems involved in emotional and social behaviours. The oxytocin (OXT) and vasopressin (AVP) systems are particularly sensitive to environmental influences during the perinatal period, a developmental window characterized by high neural plasticity. However, the long‐term effects of perinatal environmental enrichment (PEE) on hypothalamic OXT and AVP neuroplasticity and their relationship with emotional behaviour remain poorly understood. Therefore, this study investigated whether PEE induces persistent behavioural, structural, and neurochemical changes in OXT and AVP neurons in male and female mice. Male and female mice reared in either PEE or non‐enriched environment (NE) conditions were evaluated in adulthood using the elevated plus maze, light/dark box, and sociability/social novelty preference tests. Morphometric analyses assessed soma and nuclear size, as well as OXT and AVP immunoreactivity in neurons of the paraventricular nucleus (PVN) of the hypothalamus. PEE reduced anxiety‐like behaviour, as evidenced by increased open‐arm exploration in the elevated plus maze, particularly in males, and reduced latency to enter the light compartment in the light/dark box. These behavioural effects were accompanied by reduced soma and nuclear dimensions of AVP neurons in PEE males, as well as increased nuclear size and OXT immunoreactivity in both sexes. Correlation analyses showed that smaller AVP neuronal profiles were associated with reduced anxiety‐related behavioural measures, whereas larger OXT neuronal dimensions were associated with reduced risk‐assessment behaviours. Perinatal environmental enrichment induces long‐lasting, sex‐dependent structural and neurochemical plasticity in hypothalamic OXT and AVP systems, which may contribute to the enduring modulation of anxiety‐related behaviours.

## Graphical abstract

The experimental design and key findings of the study. Mice were exposed to a Perinatal enriched environment (PEE) or a Non‐enriched environment (NE) from the prenatal period through weaning (PND21). Behavioural assessments were conducted in adulthood (PND60), including the elevated plus maze, sociability test, light/dark box, sociability test and social novelty preference test. Immunofluorescence analysis was performed to assess the morphology and immunoreactivity of AVP and OXT‐expressing neurons in the paraventricular nucleus (PVN). Results demonstrate that PEE significantly alters the neurochemical profile within the PVN, evidenced by a decrease in AVP soma and nuclear area, and increased OXT immunoreactivity. Functionally, these neuroendocrine changes are associated with an anxiolytic‐like phenotype and enhanced exploratory behavior in the adult offspring, suggesting that early‐life environmental enrichment induces long‐lasting neurobehavioral and neuroendocrine adaptations.

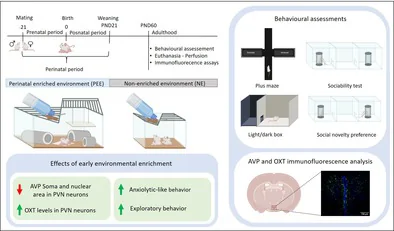

## 1 INTRODUCTION

Oxytocin (OXT) and vasopressin (AVP) are neuropeptides predominantly synthesized and secreted by magnocellular neurons located in the hypothalamic supraoptic nucleus and magnocellular and parvocellular neurons located on the paraventricular nucleus (PVN).^1^ The PVN is a major integrative hypothalamic nucleus involved in coordinating complex behavioural, reproductive, stress, autonomic, neuroendocrine and cardiovascular responses.^2^ When released as neurotransmitters, OXT is associated with social bonding, parental care, and anxiolytic effects,^3^, ^4^ while AVP is linked to increased aggression, territoriality, and stress reactivity.^5^, ^6^ These functions are interdependent, as both neuropeptidergic systems dynamically interact to influence social and emotional behaviours.^7^, ^8^, ^9^ The development of the oxytocinergic and vasopressinergic systems begins in the prenatal period and is crucial for neurodevelopment, contributing to the organization of neural circuits and influencing neural plasticity and behavioural networks.^10^ After birth, both systems continue to mature, undergoing structural and functional changes influenced by sex‐related and environmental factors.^10^, ^11^, ^12^

The perinatal period involves intense central nervous system development and remodelling, including cell proliferation, neuronal migration, synaptogenesis, and myelination, and represents a critical window during which the brain is highly sensitive to the environment.^13^ Thus, positive or negative perinatal experiences can shape neural organization and have lasting effects on stress reactivity, sociability, and the risk of neuropsychiatric disorders.^14^ Environmental enrichment (EE), by providing sensory, cognitive, and social stimuli that encourage natural behaviours like exploration, nesting, and foraging, contrasts with the sensory and social deprivation of standard housing and represents a beneficial influence during this period.^15^, ^16^

Although some studies have shown that various EE protocols modulate AVP and OXT systems,^17^, ^18^, ^19^, ^20^ little is known about the effects of perinatal environmental enrichment (PEE) specifically on the morphological characteristics of PVN AVP and OXT neurons, or about its potential association with changes in anxiety‐like and social behaviour features. Therefore, investigating PEE is important given the central roles of these neuropeptidergic systems in anxiety and social behaviour and their potential to link early experience with later outcomes. We hypothesized that PEE would induce persistent behavioural changes in adulthood accompanied by structural alterations in these neuropeptidergic systems, thereby providing a potential mechanism underlying the long‐term effects of early‐life environmental enrichment on emotional and social regulation. Thus, this study aimed to investigate the effects of PEE on behavioural outcomes, morphometric plasticity of PVN AVP and OXT neurons, and the correlations between these behavioural and morphometric parameters.

## 2 MATERIALS AND METHODS

### 2.1 Animals and experimental design

Swiss mice were obtained from the Rodent Breeding Facility of the Federal Rural University of Rio de Janeiro (UFRRJ). Sixteen breeding pairs (~60 days old) were housed under controlled temperature (21 ± 2°C) and an inverted 12 h light/dark cycle (lights on at 18:00 h), with food and water ad libitum. Breeding pairs were allocated to either a non‐enriched environment (NE; *n* = 9) or a perinatal enriched environment (PEE; *n* = 7), as described below. After 7 days of cohabitation, males were euthanized, and females remained in their respective housing conditions throughout gestation and lactation. On postnatal day 1 (PND1), litters were standardized to a maximum of eight pups (four males and four females per dam whenever possible). Thus, offspring were exposed to their respective housing conditions throughout the perinatal period, including gestation and lactation.

At postnatal day (PND) 21, pups were weaned, sexed, and initially housed with same‐sex littermates. Litter size at birth was recorded for all dams before litter standardization. At PND28, approximately half of the offspring from each litter were allocated to a separate juvenile experiment. The remaining animals were subsequently regrouped with same‐sex animals from other litters belonging to the same experimental condition (NE or PEE) to maintain groups of four animals per cage whenever possible. Due to natural variation in litter size, complete regrouping was not possible in one PEE male cage (2 animals) and one PEE female cage (3 animals). Animals from a litter NE (2 males and 2 females) were regrouped with age‐matched animals from another NE litter that was not included in the present adult analyses, allowing all NE cages to contain four animals. A detailed description of litter composition and post‐weaning cage allocation is provided in Tables S1 and S2. Following weaning, all offspring, regardless of their perinatal housing condition (NE or PEE), were maintained under identical non‐enriched housing conditions until adulthood (PND60), when behavioural testing began. Thus, the environmental manipulation differed during the perinatal period (gestation and lactation).

Behavioural assessments were conducted using the elevated plus maze (EPM), light/dark box (LDB), and sociability/social novelty preference tests. The final sample sizes were 17 NE males, 18 NE females, 14 PEE males, and 13 PEE females. Following behavioural testing, animals were deeply anesthetized and transcardially perfused for brain collection.

**FIGURE 1:**
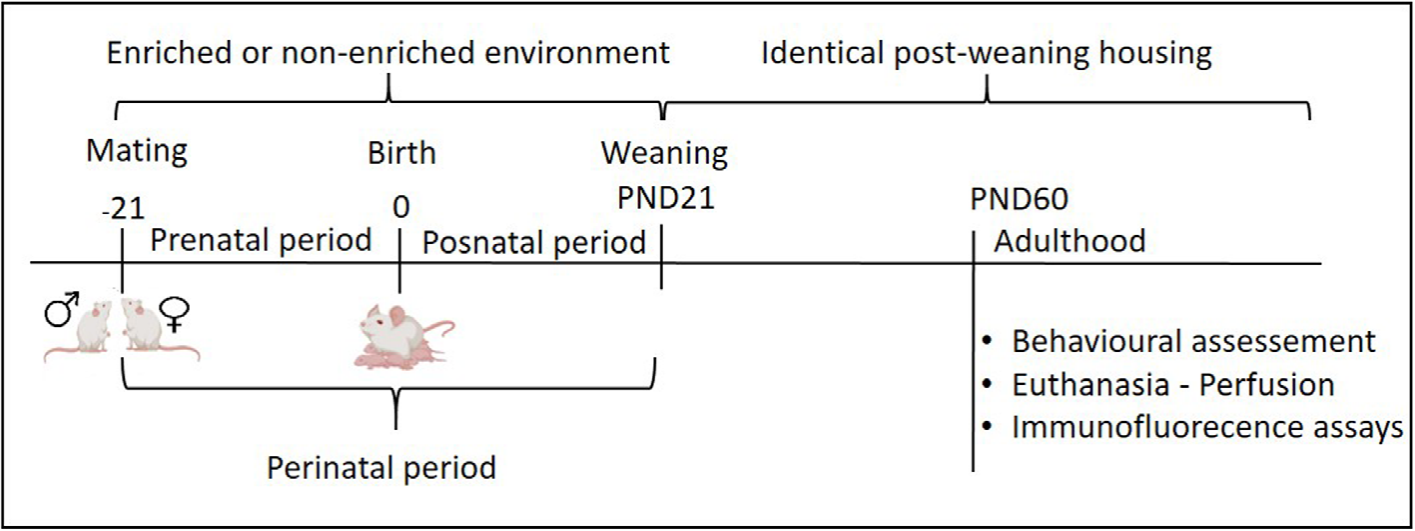
Experimental design and timeline.

The experimental protocol and the study timeline are illustrated in Figure 1.

All experimental procedures were approved by the Animal Ethics Committee of the Federal Rural University of Rio de Janeiro (CEUA‐ICBS; Protocol No. 08/2022) and were conducted in accordance with institutional guidelines and the regulations of the Brazilian National Council for the Control of Animal Experimentation (CONCEA).

### 2.2 Enrichment and non‐enriched house conditions

Animals were reared under either a PEE or a NE. The PEE protocol followed Silva‐Almeida et al.^21^ and was designed to provide stable multisensory stimulation while avoiding overexposure. The enriched cage consisted of a polypropylene box (40 L × 34 W × 20 H cm) with 2 cm wood‐shaving bedding. It contained a fixed acrylic platform (15.5 × 13.5 cm) 4.5 cm below the lid, allowing external visual exploration, along with a tunnel (7 × 7 cm), arch (11.5 × 4.5 cm), circular shelter (16 × 7 cm, closed at one end), and paper towels for nesting. Running wheels were omitted to avoid confounding effects of physical exercise. Control animals were housed in standard opaque cages (30 × 20 × 13 cm) with 2 cm wood‐shaving bedding.

The NE group were housed in standard opaque polypropylene cages (30 × 20 × 13 cm) containing 2 cm of wood‐shaving bedding only, without nesting material or additional environmental enrichment. This terminology (non‐enriched environment) does not imply a validated model of limited nesting or maternal deprivation, but rather describes a housing condition lacking additional environmental enrichment.

### 2.3 Behavioural tests

All behavioural sessions were video‐recorded for later analysis. Behavioural scoring was performed manually by a single trained experimenter who was blinded to the experimental groups throughout data collection and analysis. Behavioural events were scored according to predefined and standardized criteria that were applied consistently across all animals. To ensure consistency, representative behavioural recordings were independently re‐evaluated by the same blinded experimenter using the predefined scoring criteria. Tests were conducted in the following order: elevated plus maze (EPM), light/dark box (LDB), and sociability/social novelty preference tests. Testing began at adulthood (PND60) and was conducted during the dark phase, with 48‐h intervals between consecutive tests.^21^ Animals were euthanized and transcardially perfused on the day after the last behavioural test.

#### 2.3.1 Elevated plus maze

The apparatus consisted of an opaque acrylic platform elevated 45 cm above the floor, with four arms arranged in a plus configuration: two open arms without walls and two closed arms with 25 cm high walls, connected by a central platform. Light intensity was set to 200 ± 10 lux in open arms and 20 ± 2 lux in closed arms. Each mouse was placed on the central platform and allowed to freely explore for 5 min. Anxiety‐related parameters included the percentage of time spent in and number of entries into the open arms, and number of risk‐assessments behaviours. The risk assessment behaviour was quantified as the combined frequency of two ethologically distinct behaviours: stretch‐attend postures (defined as the animal extending its body from a closed arm into on the open arms while keeping its hind paws in the closed arm), and head‐dipping (defined as lowering the head over the edge of the open arm towards the floor below). Locomotor activity was evaluated by the total number of closed‐arm entries.^22^, ^23^, ^24^

#### 2.3.2 Light/dark box

The apparatus consisted of two interconnected compartments: a brightly lit white chamber (26.5 × 26 × 20 cm; 400 ± 10 lux) and a dark‐black‐walled chamber (26.5 × 17.5 × 20 cm; ≤4 lux). A small opening allowed free movement between compartments. Each mouse was placed in the dark chamber at the beginning of the test and allowed to freely explore the apparatus for 5 min while behaviour was recorded. Parameters analysed included the time spent and number of crossings into the light compartment, latency to first enter the light area, latency to return to the dark area, total light‐zone crossings, number of transitions between compartments, and number of risk‐assessment behaviours. The risk‐assessment behaviour was quantified as the combined frequency of stretch‐attend postures (defined as the animal extended its body from the dark into the light compartment while keeping its hind paws in the dark zone) and scanning behaviour (defined as brief, sustained head orientations towards the light compartment while remaining within the protected dark area). These behaviours were aggregated into a single risk assessment metric to capture the animal's decision‐making conflict between exploration and avoidance.^25^

#### 2.3.3 Sociability and social novelty preference tests

Social behaviour was evaluated in a three‐chamber apparatus (20 × 44 × 35 cm) using a procedure adapted from Crawley and Moy et al.^26^, ^27^ The apparatus consisted of an acrylic box with three chambers (20 × 44 × 35 cm) divided by walls with rectangular openings allowing free movement between compartments. In each lateral chamber, a cylindrical wire cage (11 cm high × 10.5 cm in diameter) was placed. The test comprised three phases: (1) Habituation (5 min, session 0)—the mouse freely explored all chambers without social stimuli; (2) Sociability (10 min, session 1)—an unfamiliar same‐sex conspecific (‘stranger 1’) was placed inside a wire cage (11 × 10.5 cm Ø) in one lateral chamber, while the opposite cage remained empty; (3) Social Novelty Preference (10 min, session 2)—a second unfamiliar conspecific (‘stranger 2’) was introduced into the previously empty cage. The stimulus animals (‘strangers’) were age‐matched, same‐sex mice housed under standard conditions, and had no prior contact (visual, olfactory, or auditory) with the experimental animals. Parameters analysed included interaction time with each stimulus (stranger 1, stranger 2, and empty cage), time spent in the central chamber, grooming duration, and the number of rearings (defined as the animal standing on its hind limbs, with or without forepaw support against the wall of the apparatus). For statistical analysis, the time spent interacting with the conspecific enclosed in the wire cage was measured and converted into preference indices defined as^28^:

$$\frac{\text{Time with stranger}\;1}{\text{Time with stranger}\;1+\text{Time with empty cage}}\times 100\;\left(\text{session}\;1\right),$$

and

$$\frac{\text{Time with stranger}\;2}{\text{Time with stranger}\;2+\text{Time with stranger}\;1}\times 100\;\left(\text{session}\;2\right).$$

### 2.4 Euthanasia and brain processing

On the day following the last behavioural test, animals were anesthetized with sodium thiopental (100 mg/kg, i.p.) and perfused transcardially with heparinized saline followed by 4% paraformaldehyde (PFA) in PBS. Brains were collected, post‐fixed in 4% PFA, cryoprotected in 30% sucrose, and coronally sectioned at 30 μm using a cryostat. Sections containing the paraventricular nucleus (PVN; −0.70 to −1.22 mm)^29^ were stored for immunofluorescence analysis.

### 2.5 Immunohistochemistry of AVP+ and OXT+ PVN neurons

Immunohistochemistry was performed to evaluate morphometric parameters of the soma and nuclei of oxytocinergic and vasopressinergic neurons in the PVN, using double labelling for OXT and AVP. Free‐floating sections were first washed in 0.01 M PBS (three times for 5 min each) under gentle agitation at room temperature. Sections were then incubated for 1 h in blocking solution containing 5% normal donkey serum (NDS) and 0.3% Triton X‐100 in 0.1 M PBS. Subsequently, sections were incubated overnight at 4°C with primary antibodies diluted in incubation solution (2% NDS and 0.3% Triton X‐100 in 0.1 M PBS), including rabbit anti‐oxytocin (Peninsula Laboratories, T‐4084, AB_518524; 1:25,000) and guinea pig anti‐(Arg8)‐vasopressin (Peninsula Laboratories, T‐5048, AB_518680; 1:25,000). After washing in 0.01 M PBS, sections were incubated for 1 h at room temperature with the appropriate secondary antibodies: Alexa Fluor 488‐conjugated donkey anti‐rabbit IgG (H + L) (Jackson ImmunoResearch, 711–545‐152, AB_2340620; 1:300) and Alexa Fluor 594‐conjugated donkey anti‐guinea pig IgG (H + L) (Jackson ImmunoResearch, 711–545‐152, AB_2337442; 1:300), diluted in incubation solution. Following additional washes, sections were incubated with DAPI for 20 min at room temperature. Sections were then mounted on slides, air‐dried, coverslipped with Fluoromount™, and sealed.

### 2.6 Image acquisition and morphometric analyses

Images were acquired using a Zeiss Celldiscoverer 7 confocal microscope (40× objective). For improved visualization during analysis, the Alexa Fluor 594 signal (AVP) was digitally pseudocoloured green. Image analysis was performed using ImageJ software (National Institutes of Health, Bethesda, MD, USA). All morphometric measurements and fluorescence quantification were performed by the same blinded investigator using standardized analysis parameters. Representative images were reanalysed by the same evaluator to verify measurement consistency. The soma and nuclear area and diameter, as well as the immunoreactive optical density of the soma, were measured in AVP and OXT‐positive neurons by manually outlining cell and nuclear boundaries using ImageJ. PVN neurons were randomly selected for analysis, and only cells displaying clearly defined DAPI‐stained nuclei and complete soma profiles were included. Up to 20 neurons per animal were analysed, and the obtained values were averaged to generate a single mean value per animal for statistical comparison. Initially, six animals per experimental group were randomly selected for neuroanatomical analyses. To minimize potential litter effects, only one animal from each litter was included. Final sample sizes ranged from four to six animals per group, as some brain sections were excluded because they did not meet the predefined quality criteria for morphometric analyses.

### 2.7 Statistical analysis

Comparisons among the four experimental groups were made with two‐way ANOVA followed by Tukey's post hoc test. For variables that do not follow normality (Shapiro–Wilk test), rank transformation was applied prior to analysis. Data are presented as boxplots (median, interquartile range, whiskers). Associations were assessed with Spearman's rank correlation, and linear regression was used to compare intercepts and slopes between sexes and treatment groups. Statistical significance was set at *p* < .05. Analyses were performed with GraphPad Prism 9.0 (GraphPad Software, San Diego, CA, USA).

## 3 RESULTS

### 3.1 Litter characteristics

Litter size at birth did not differ significantly between the NE and PEE groups (two‐tailed Mann–Whitney test, *p* = .154; Figure S1), indicating that the perinatal housing conditions did not affect litter size before litter standardization. Detailed information on litter size at birth, litter standardization, and post‐weaning cage allocation is provided in Tables S1 and S2.

### 3.2 Early‐life housing conditions influence anxiety‐like behaviour in the elevated plus maze

**FIGURE 2:**
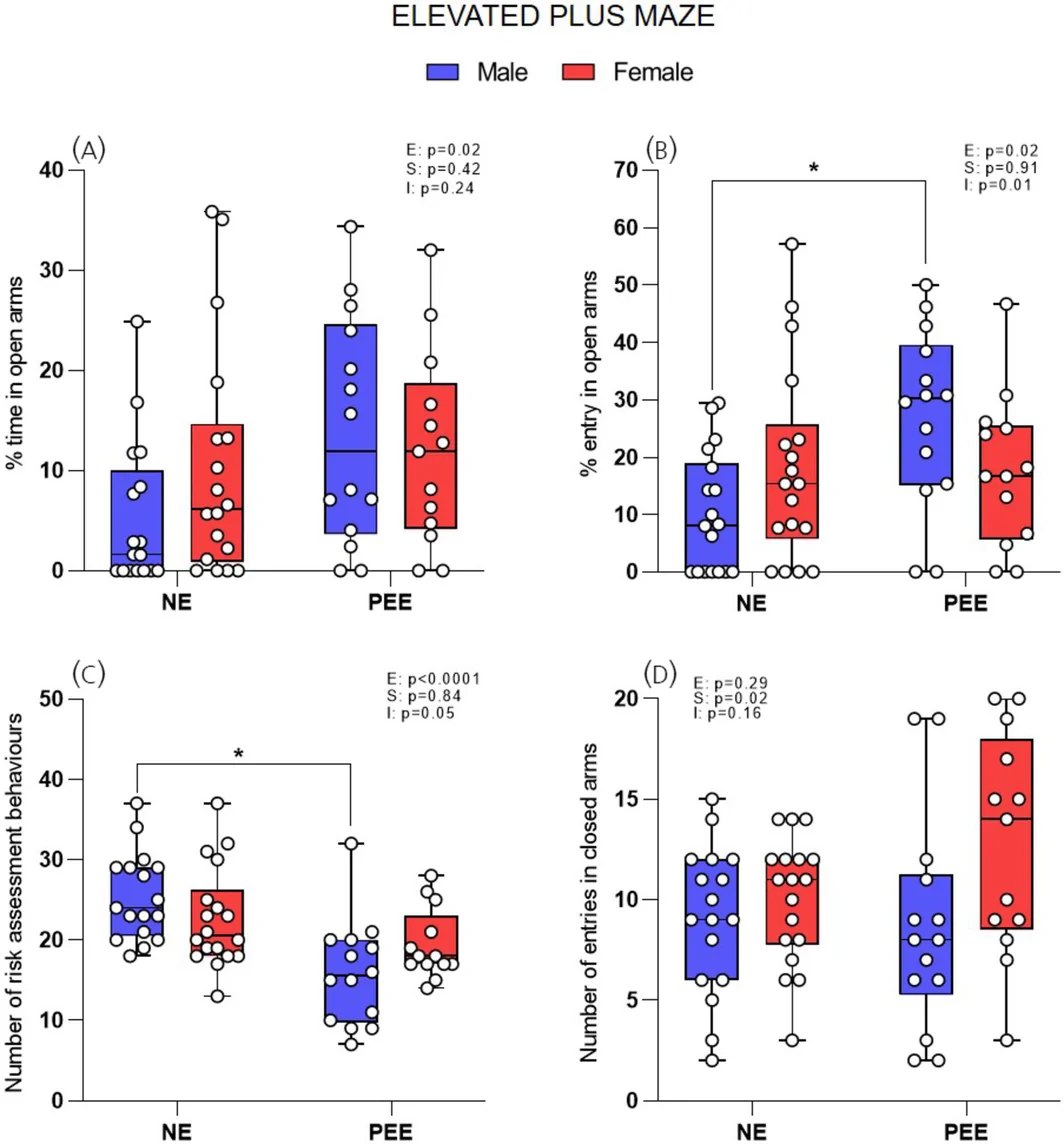
Behavioural effects of perinatal environmental enrichment in the elevated plus maze test. (A) % time in open arms; (B) % entry in open arms; (C) number of risk assessment behaviours; and (D) number of entries in closed arms. Data for panels A and B were rank‐transformed prior to ANOVA. *n* = 13–18. Significant Tukey's post hoc test: *p < .05. Two‐way ANOVA results are shown in the inset panel. E: main effect of environment; S: main effect of sex; I: interaction; NE: non‐enriched environment; PEE: perinatal enriched environment. Data are presented as boxplots showing the median, interquartile range, whiskers, and individual data points.

In the EPM, relative time in the open arms was affected by environment (*F*_(1,58)_ = 5.17, *p* = .02), with PEE mice spending more time there than NE mice (Figure 2A). Open‐arm entry frequency showed an environment × sex interaction (*F*_(1,58)_ = 5.78, *p* = .01) and a main effect of environment (*F*_(1,58)_ = 5.64, *p* = .02); post hoc test indicated more open‐arm entries in PEE males than NE males (*p* < .01) (Figure 2B). Risk‐assessment behaviours showed an environment × sex interaction (*F*_(1,58)_ = 4.52, *p* = .03) and a main effect of environment (*F*_(1,58)_ = 18.94, *p* < .0001); PEE animals displayed fewer risk‐assessment behaviours than NE animals, with PEE males showing fewer than NE males (*p* < .0001) and NE females (*p* < .01) (Figure 2C). Sex affected closed‐arm entries (*F*_(1,58)_ = 5.09, *p* = .02), with females making more entries than males (Figure 2D).

Taken together, these findings indicate that animals reared in the enriched environment exhibited reduced anxiety‐like behaviour compared with those reared in the non‐enriched environment, particularly in males, as evidenced by increased exploration of the open arms and reduced risk‐assessment behaviours.

### 3.3 Early‐life housing conditions influence anxiety‐like behaviour in the light/dark box

**FIGURE 3:**
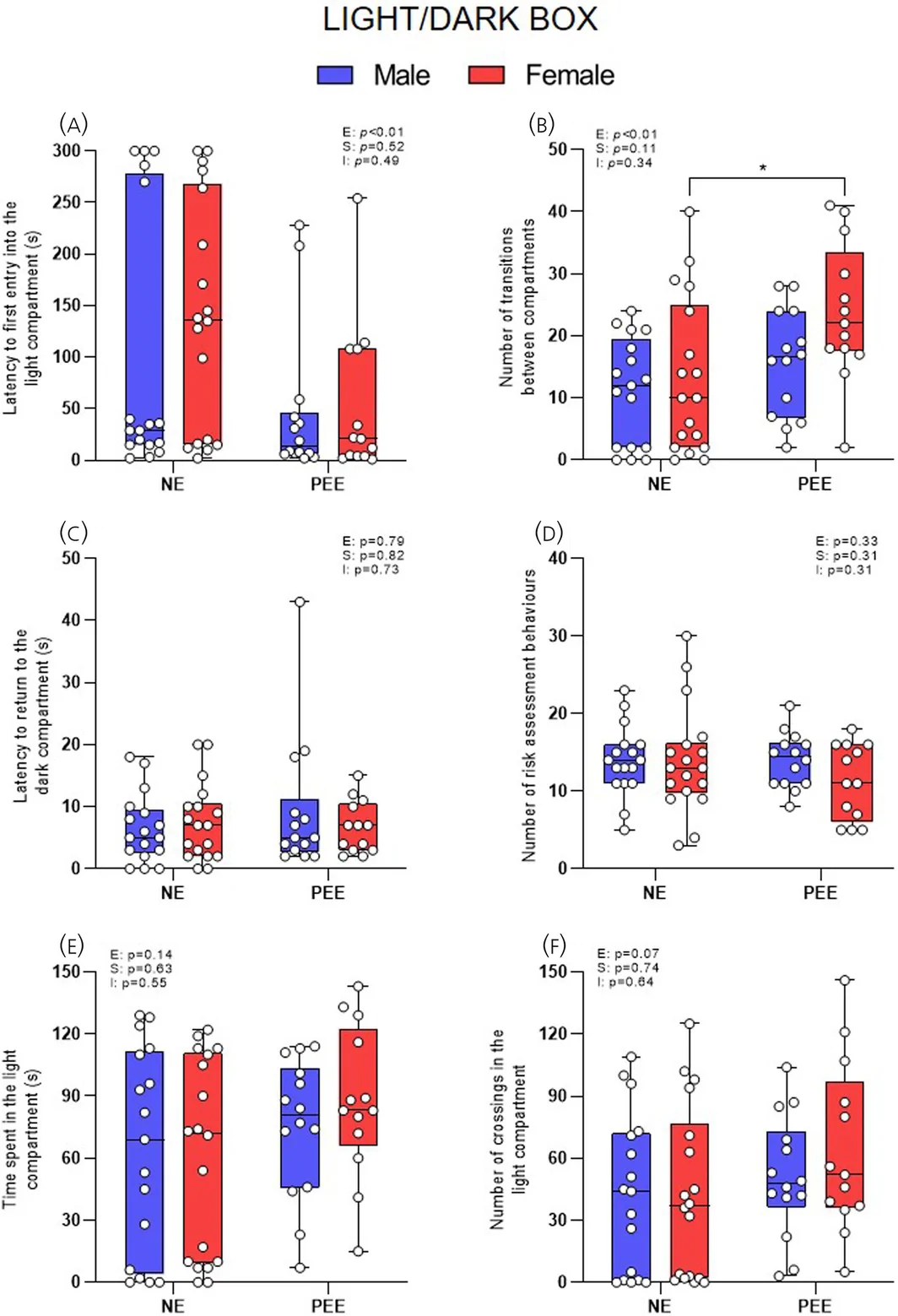
Behavioural effects of perinatal environmental enrichment in the light–dark box test. (A) latency to first entry into the light compartment (S); (B) number of transitions between compartments; (C) latency to return to the dark compartment (s); (D) number of risk assessment behaviours; (E) time spent in the light compartment (s); and (F) number of crossings in the light compartment. Data for panels A, B, C, E, and F were rank‐transformed prior to ANOVA. *n* = 13–18. Significant Tukey's post hoc test: *p < .05. Two‐way ANOVA results are shown in the inset panel. E: main effect of environment; S: main effect of sex; I: interaction; NE non‐enriched environment; PEE: perinatal enriched environment. Data are presented as boxplots showing the median, interquartile range, whiskers, and individual data points.

In the LDB, latency to enter the light compartment was affected by environment (*F*_(1,58)_ = 7.56, *p* < .01), with PEE mice entering sooner than NE mice (Figure 3A). Transitions between compartments showed a main effect of environment (*F*_(1,58)_ = 9.19, *p* < .01), with PEE mice making more transitions than NE mice. Post hoc test indicated PEE females made more transitions than NE males (*p* = .01) and NE females (*p* = .03), but did not differ from PEE males (Figure 3B). No significant effects or interactions were found for latency to return to the dark compartment (Figure 3C), number of risk assessments (Figure 3D), time spent in the light compartment (Figure 3E) or number of crossings in the light area (Figure 3F).

Together, these results indicate that early‐life housing conditions influenced anxiety‐like behaviour, with animals reared in the enriched environment displaying reduced hesitation to explore the light compartment and increased exploratory behaviour compared with those reared in the non‐enriched environment.

### 3.4 Early‐life housing conditions increase exploratory behaviour without affecting sociability

**FIGURE 4:**
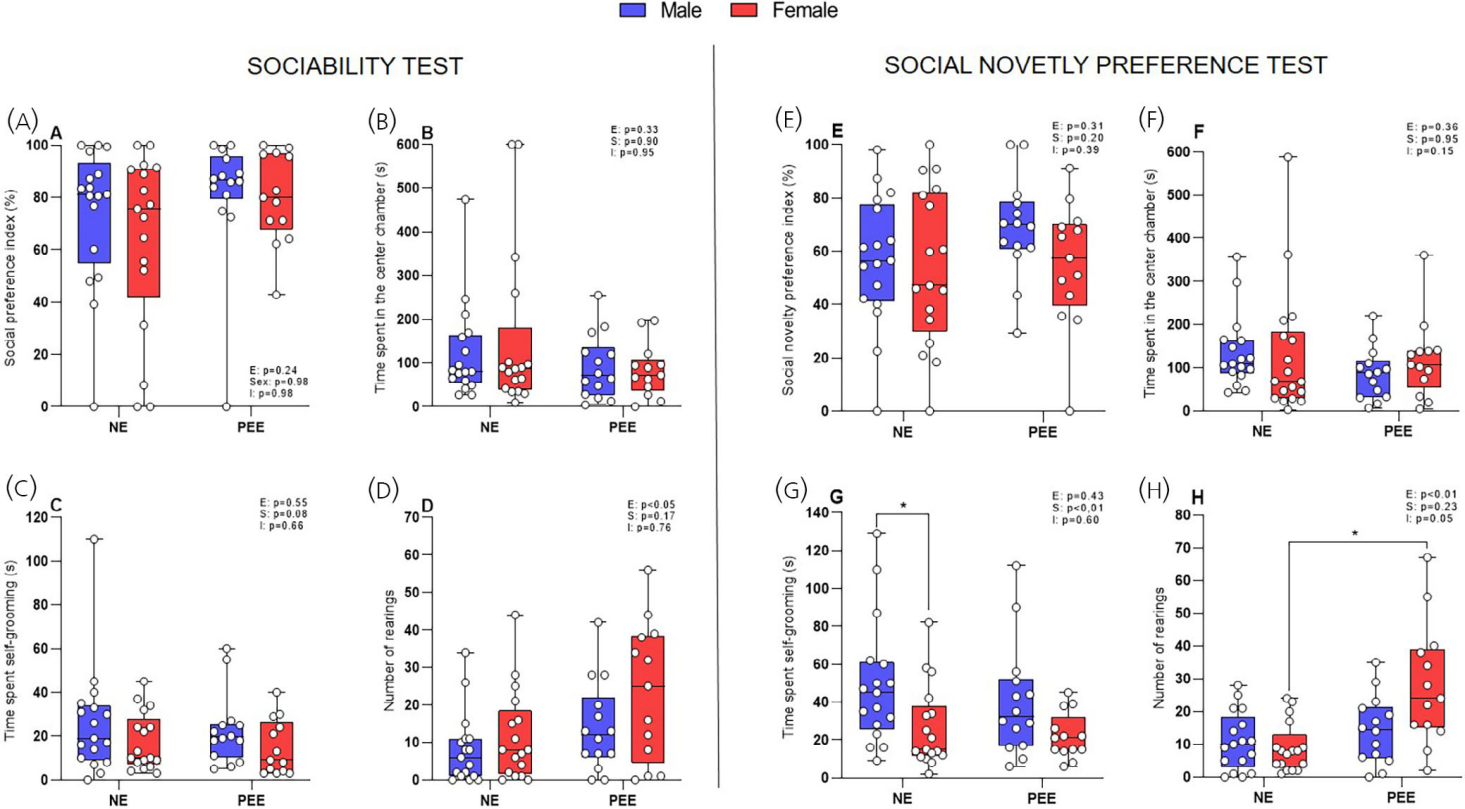
Behavioural effects of perinatal environmental enrichment in the sociability and social novelty preference tests (sessions 1 and 2). Panels A–D show data from session 1: (A) social preference index (%); (B) time spent in the centre chamber (S); (C) time spent self‐grooming (s); and (D) number of rearings. Panels E–H show data from session 2: (E) social novelty preference index (%); (F) time spent in the centre chamber (s); (G) time spent self‐grooming (s); and (H) number of rearings. Data for panels B, C, D, E, F and G were rank‐transformed prior to ANOVA. *n* = 13–17 (one NE female was excluded from analysis due to a technical issue during protocol execution). Significant Tukey's post hoc test: *p < .05. Two‐way ANOVA results are shown in the inset panel. E: main effect of environment; S: main effect of sex; I: Interaction; NE non‐enriched environment; PEE: perinatal enriched environment. Data are presented as boxplots showing the median, interquartile range, whiskers, and individual data points.

In the sociability test, rearing frequency was affected by environment (*F*_(1,57)_ = 5.10, *p* = .02), with PEE animals showing more rearings than NE animals (Figure 4D). In the social‐novelty test, grooming time was affected by sex (*F*_(1,57)_ = 11.16, *p* = .001), with males grooming more than females (Figure 4G). Rearing was again also affected by environment (*F*_(1,57)_ = 10.49, *p* = .001), with higher rearing in PEE mice; post hoc analysis indicated PEE females reared more than NE males (*p* = .01) and NE females (*p* < .01) (Figure 4H). No significant effects or interactions were found for the social‐preference index or time spent in the centre chamber in session 1 (Figure 3A,B), nor for the social‐novelty index or time spent in the centre chamber in session 2 (Figure 4E,F).

Collectively, these findings indicate that PEE increased exploratory behaviour, as reflected by a higher rearing frequency, while leaving sociability and social novelty preference unchanged.

### 3.5 Early‐life housing conditions induce sex‐dependent structural plasticity in PVN AVP neurons

**FIGURE 5:**
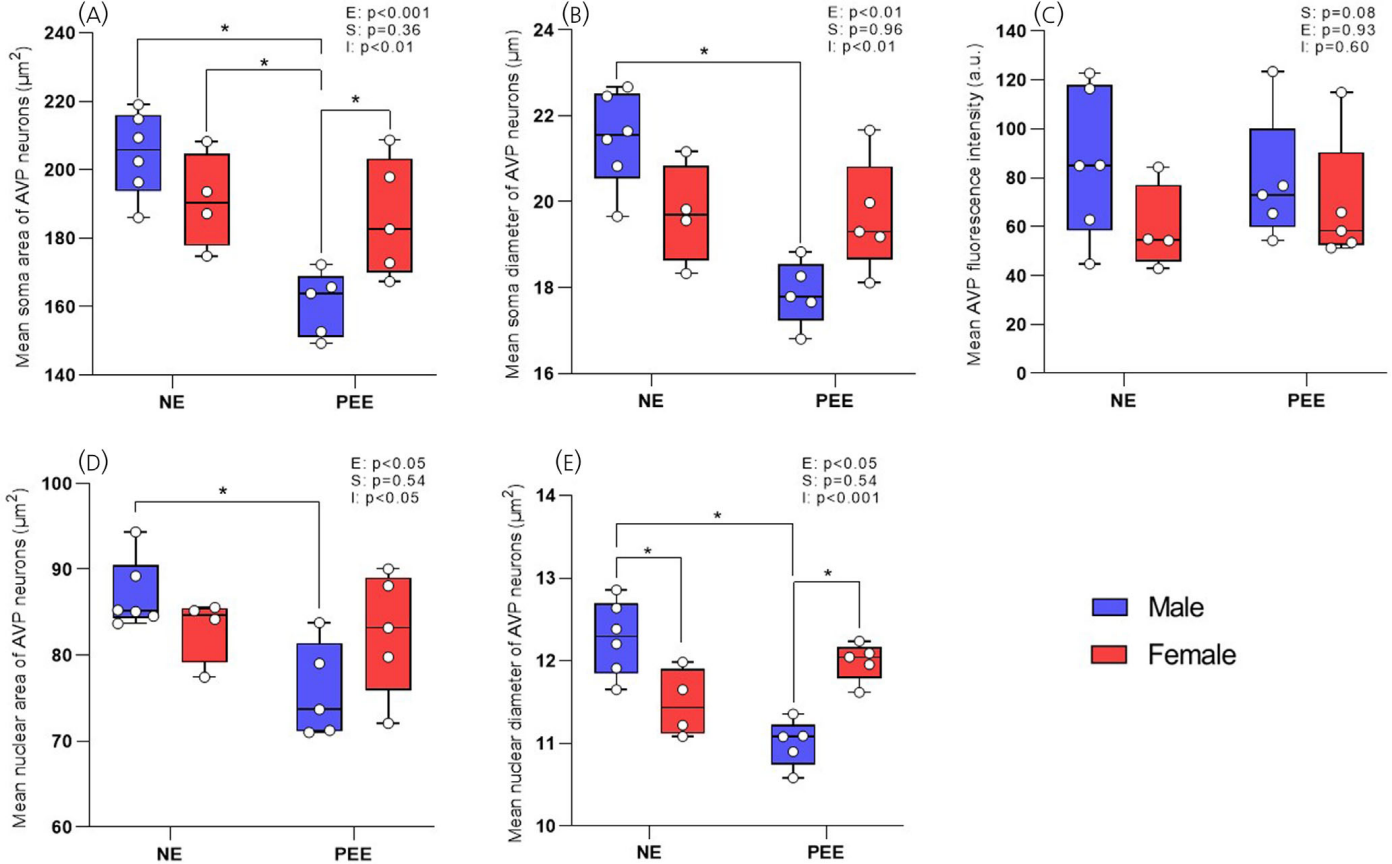
Effects of perinatal environmental enrichment on the soma and nuclei of vasopressin‐immunoreactive neurons in the PVN. (A) mean soma area of AVP neurons (μm^2^); (B) mean soma diameter of AVP neurons (μm); (C) mean AVP fluorescence intensity (a.u.); (D) mean nuclear area of AVP neurons; and (E) mean nuclear diameter of AVP neurons (μm). The data for panel B were rank‐transformed prior to ANOVA. *n* = 4–6. Significant Tukey's post hoc test: *p < .05. Two‐way ANOVA results are shown in the inset panel. E: main effect of environment; S: main effect of sex; I: interaction; NE non‐enriched environment; PEE: perinatal enriched environment. Data are presented as boxplots showing the median, interquartile range, whiskers, and individual data points.

In the PVN, analysis of vasopressinergic neurons revealed a significant interaction between sex and environment (*F*_(1,16)_ = 10.24, *p* < .01) for mean soma area, and a main effect of environment (*F*_(1,16)_ = 16.27, *p* = .001), with PEE animals showing smaller soma areas than NE animals. Post hoc analysis showed that PEE males had smaller soma areas than NE males (*p* < .001), NE females (*p* = .01), and PEE females (*p* = .04) (Figure 5A). Soma diameter also showed a significant interaction (*F*_(1,16)_ = 12.49, *p* < .01) and a main effect of environment (*F*_(1,16)_ = 13.55, *p* < .01), with PEE animals showing smaller soma diameters. Post hoc tests indicated that PEE males showed smaller soma diameters than NE males (*p* < .001) (Figure 5B).

**FIGURE 6:**
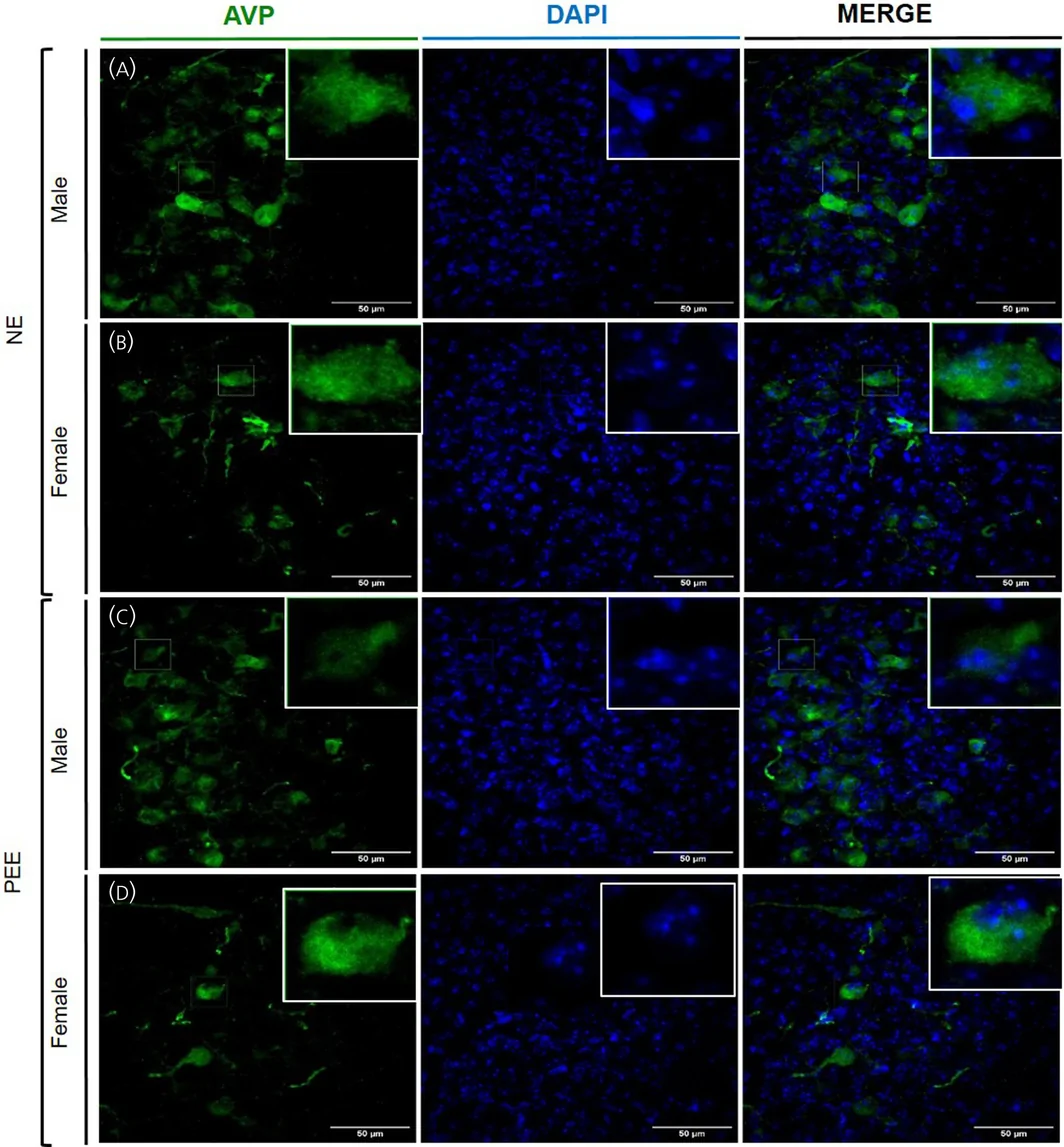
Representative photomicrographs of coronal PVN sections illustrating AVP‐positive neurons (green) and nuclei (blue) from NE males (A), NE females (B), PEE males (C), and PEE females (D), scale bar: 50 μm. NE: non‐enriched environment; PEE: perinatal enriched environment.

For nuclear parameters, mean nuclear area showed a significant interaction between sex and environment (*F*_(1,16)_ = 5.04, *p* = .03), and effect of environment (*F*_(1,16)_ = 5.95, *p* = .02), with PEE animals showing smaller nuclear areas. Post hoc analysis showed that PEE males had smaller nuclear areas than NE males (*p* = .01) (Figure 5D). Nuclear diameter analysis revealed a significant interaction between factors (*F*_(1,16)_ = 19.81, *p* < .001) and a main effect of environment (*F*_(1,16)_ = 6.41, *p* = .02), with PEE animals showing smaller nuclear diameters. Post hoc tests indicated that PEE males had smaller nuclear diameters than NE males (*p* < .01) and PEE females (*p* = .02) (Figure 5E). And NE males had smaller nuclear diameters than NE females (*p* = .01). Representative images of PVN vasopressinergic neurons for each group are shown in Figure 6.

Collectively, these findings demonstrate marked differences in AVP neuronal morphology between animals reared in enriched and non‐enriched environments, with the effects being more pronounced in males, indicating sex‐dependent structural plasticity associated with early‐life housing conditions.

### 3.6 PEE enhances structural and neurochemical plasticity in PVN OXT neurons

**FIGURE 7:**
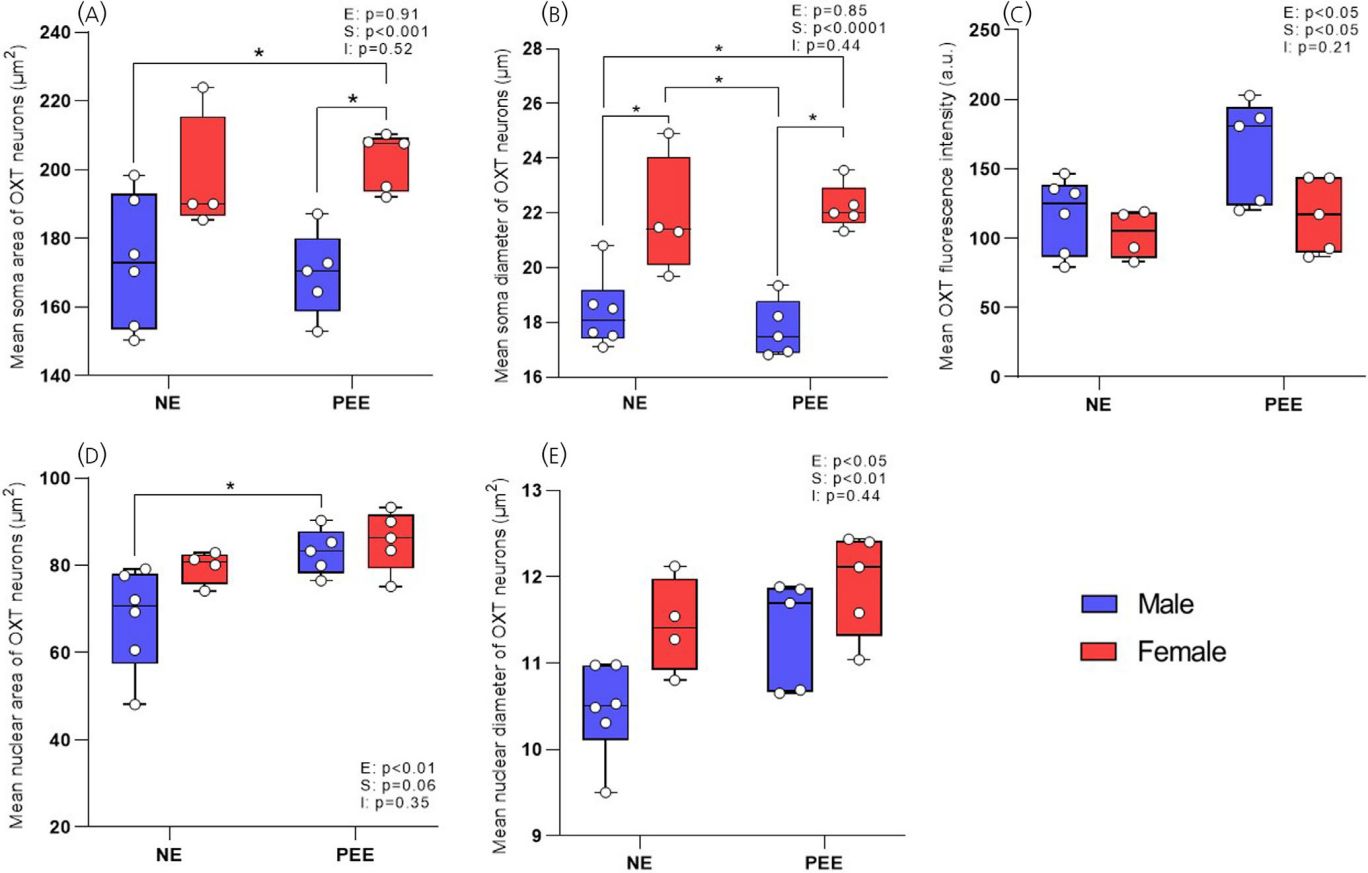
(A) mean soma area of OXT neurons (μm^2^); (B) mean soma diameter of OXT neurons (μm); (C) mean OXT fluorescence intensity (a.u.); (D) mean nuclear area of OXT neurons; and (E) mean nuclear diameter of OXT neurons (μm). *n* = 4–6. Significant Tukey's post hoc test: *p < .05. Two‐way ANOVA results are shown in the inset panel. E: main effect of environment; S: main effect of sex; I: interaction; NE: non‐enriched environment; PEE: perinatal enriched environment. Data are presented as boxplots showing the median, interquartile range, whiskers, and individual data points.

Analysis of oxytocinergic neurons revealed a significant main effect of sex on soma area (*F*_(1,16)_ = 17.22, *p* < .001), with females exhibiting larger somata than males. Post hoc tests showed that PEE females had larger soma areas than NE males (*p* = .02) and PEE males (*p* = .01) but did not differ from NE females (*p* = .95) (Figure 7A). Soma diameter was also significantly affected by sex (*F*_(1,16)_ = 40.57, *p* < .0001), with females showing larger soma diameters than males. Post hoc analysis revealed that PEE females exhibited greater diameters than NE males (*p* = .001) and PEE males (*p* < .001), but did not differ from NE females (*p* = .97) (Figure 7B). Analysis of the OXT immunoreactive optical density in the soma revealed significant main effects for both environment (*F*_(1,16)_ = 5.42, *p* = .03) and sex (*F*_(1,16)_ = 5.45, *p* = .03). Specifically, EE animals exhibited higher OXT levels compared to standard‐housed animals, while females displayed lower OXT levels than males (Figure 7C).

**FIGURE 8:**
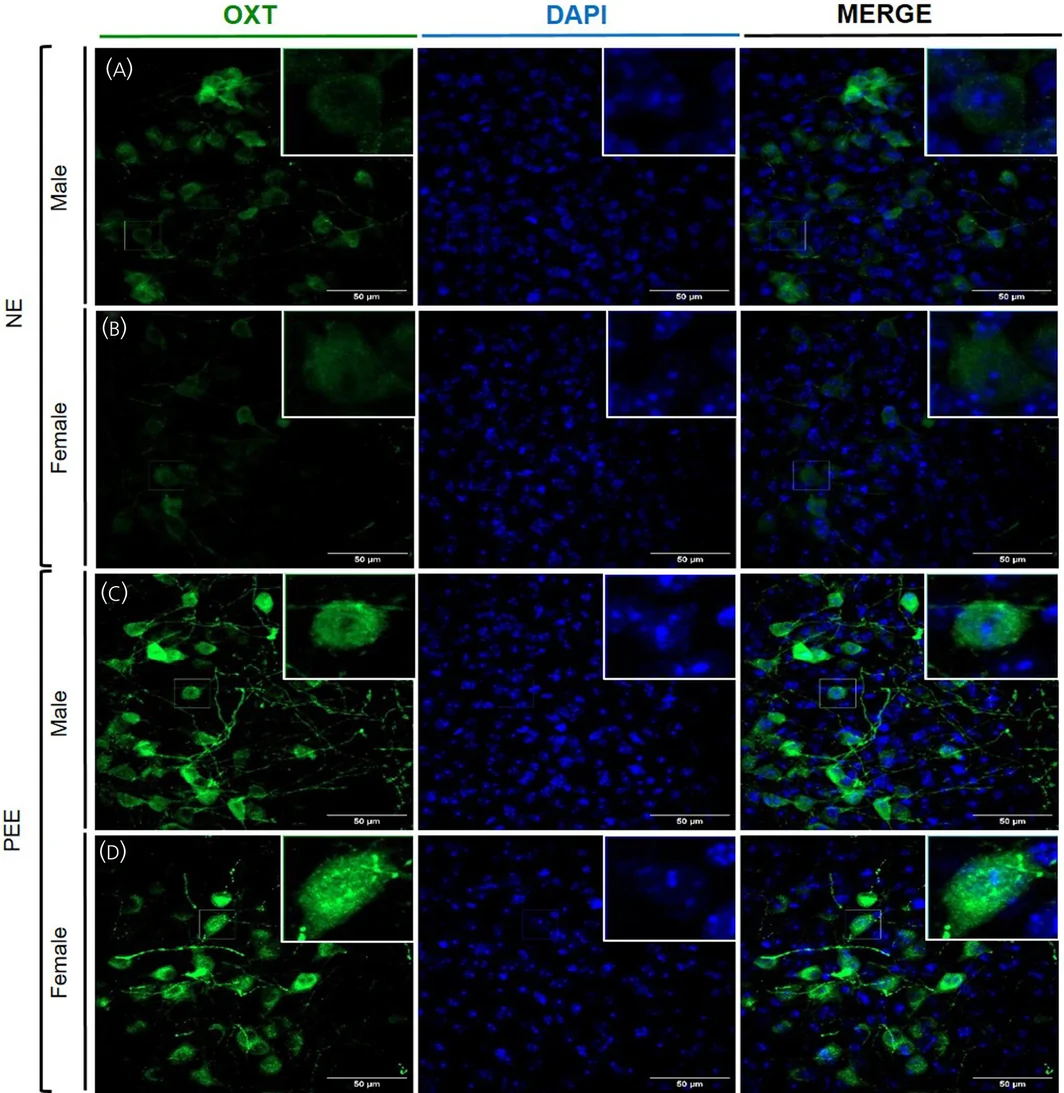
Representative photomicrographs of coronal PVN sections illustrating OXT‐positive neurons (green) and nuclei (blue) from NE males (A), NE females (B), PEE males (C), and PEE females (D), scale bar: 50 μm. NE: non‐enriched environment; PEE: perinatal enriched environment.

Nuclear area (*F*_(1,16)_ = 12.09, *p* < .01) and diameter (*F*_(1,16)_ = 6.80, *p* = .01) were both significantly affected by environment, with PEE animals showing larger. Post hoc tests indicated that PEE males had a larger nucleus area than NE males (*p* = .02). Sex significantly influenced nuclear diameter (*F*_(1,16)_ = 8.55, *p* = .009), with females exhibiting greater mean diameter values (Figure 7D,E). Representative images of PVN oxytocinergic neurons for each group are shown in Figure 8.

Taken together, these results demonstrate that early‐life housing conditions were associated with structural and neurochemical plasticity in hypothalamic OXT neurons, characterized by increased nuclear size and OXT immunoreactivity in animals reared in the enriched environment, whereas sex differences were predominantly observed in soma morphology.

### 3.7 Hypothalamic neuronal morphology is associated with anxiety‐related behavioural outcomes

**FIGURE 9:**
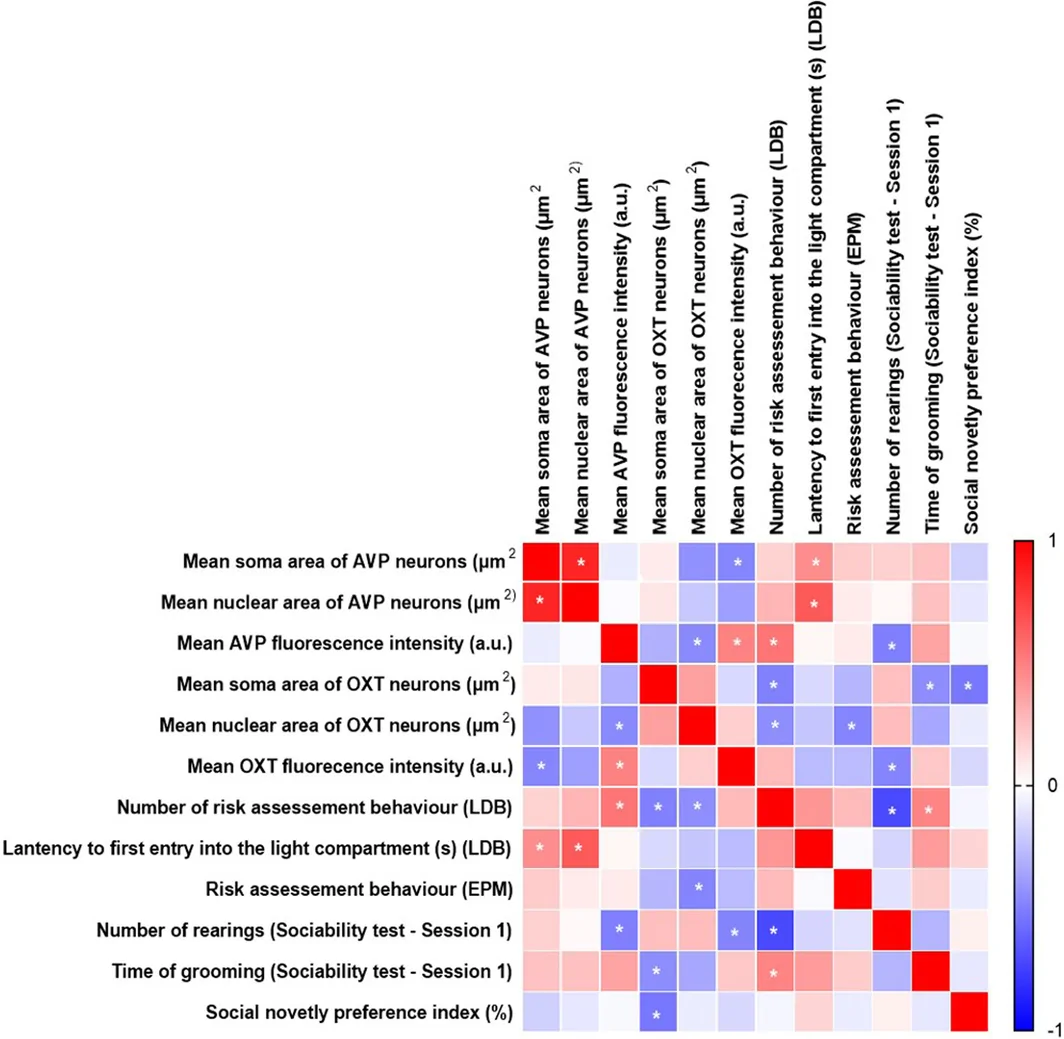
Spearman correlation matrix between immunofluorescence parameters and behavioural test variables. *p < .05. EPM: elevated plus maze, LDB: light/dark box.

Significant correlations were found between behavioural and morphological parameters. The mean soma and nuclear areas of AVP neurons were positively correlated with latency to first entry into the light compartment in the LDB test (*p* = .04 and *p* < .01, respectively). Mean AVP fluorescence intensity was positively correlated with the number of risk assessment behaviours in the LDB (*p* = .01) and negatively correlated with the number of rearings in the sociability test (session 1) (*p* = .02). For OXT neurons, mean soma area was negatively correlated with the number of risk assessments in the LDB (*p* = .02) and grooming time in the sociability test (*p* = .04). Mean nuclear area was negatively correlated with risk assessments in both the LDB (*p* = .04) and EPM (*p* = .03) (Figure 9).

Taken together, these findings suggest that structural and neurochemical changes in hypothalamic AVP and OXT neurons are associated with the long‐term modulation of anxiety‐related behaviours following early‐life housing conditions.

## 4 DISCUSSION

Early‐life environmental conditions critically shape neuroendocrine systems that regulate emotional and social behaviour. Oxytocinergic and vasopressinergic pathways are central to anxiety, sociability, and stress responsiveness, but the effects of perinatal environmental enrichment (EE) on their development remain unclear. Here we show that perinatal EE alters anxiety‐like and exploratory behaviour and produces long‐term morphological changes in OXT‐ and AVP‐producing neurons in the PVN.

In the social interaction and novelty preference tests, EE did not alter direct social preference or novelty discrimination but increased rearing frequency, suggesting enhanced exploratory activity within social contexts.^30^ These findings align with reports showing transient increases in social exploration following early EE exposure, although long‐term effects on sociability remain inconsistent.^31^, ^32^

In contrast, clear anxiolytic effects of EE were observed in the EPM, particularly in males, which showed greater open‐arm exploration and reduced risk‐assessment behaviours. Similar findings were previously observed in our laboratory using a comparable enrichment protocol.^21^ In the LDB, enriched mice exhibited shorter latencies to enter the illuminated compartment and more transitions between chambers, consistent with reduced anxiety and heightened exploratory drive.^33^, ^34^

Previous studies have reported heterogeneous outcomes depending on EE timing, complexity, and the presence of social or physical components.^20^, ^31^ Here, the use of a static, low‐instability enrichment may have minimized overstimulation while promoting adaptive exploration. Although EE's anxiolytic effects are more evident in stress‐exposed models,^35^, ^36^, ^37^ our findings indicate that even under baseline conditions, moderate perinatal EE can induce lasting behavioural benefits.

Morphological analyses revealed that early‐life environmental conditions significantly influenced both OXT and AVP neurons. In males, vasopressinergic neurons of the PVN displayed reduced soma and nuclear dimensions in the PEE group compared with the NE group, without changes in AVP fluorescence intensity. These findings are consistent with previous studies showing that early‐life environmental conditions influence vasopressinergic system development, with positive early‐life experiences generally associated with reduced vasopressin signalling and early‐life stress with increased AVP activity.^38^, ^39^, ^40^ Thus, the present findings support that early‐life environmental experiences can induce long‐lasting, sex‐dependent changes in the vasopressinergic system.

Conversely, EE increased nuclear size and enhanced OXT immunoreactivity of the OXT neurons of both sexes, in line with evidence that enriched environments enhance oxytocin synthesis and neuronal activity.^18^, ^19^, ^41^, ^42^ In contrast, early‐life adversity, such as maternal separation or immune challenge, often diminishes OXT expression.^43^, ^44^, ^45^

Sex differences were evident across multiple parameters. Females exhibited larger soma and nuclear areas but lower OXT immunoreactivity than males, independent of environmental condition. This sexual dimorphism likely reflects interactions between sex hormones and OXT system regulation.^39^, ^46^ Oestrogens are known to upregulate OXT transcription and receptor expression,^47^, ^48^ and fluctuations across the oestrous cycle can modulate PVN neuronal excitability.^49^ Although the observed sex differences were consistent, the oestrous cycle was not monitored in the present study and may have contributed to variability within the female groups. Future studies controlling for oestrous cycle stage will be important to further characterize sex‐specific effects of early‐life environmental enrichment.

Functionally, structural modifications in OXT and AVP neurons may underlie the behavioural effects of EE. Oxytocin has been linked to anxiolysis and enhanced stress coping, while vasopressin often promotes vigilance and defensive behaviours in a sex‐dependent manner.^12^, ^50^, ^51^ The inverse morphological patterns observed, OXT hypertrophy and AVP reduction, support a neuroendocrine shift towards reduced anxiety and increased behavioural adaptability.^7^, ^51^

Correlation analyses further supported these functional associations between neuronal morphology and behaviour. Larger AVP neurons were associated with increased anxiety‐like measures, whereas larger OXT neurons were associated with reduced risk‐assessment behaviours and grooming, reinforcing the relationship between hypothalamic structural plasticity and long‐term behavioural outcomes. However, regression analyses stratified by sex and environmental condition were not significant, suggesting that these associations are influenced by multiple interacting biological factors rather than simple linear relationships.

The mechanisms mediating the effects of perinatal environmental enrichment are likely multifactorial. Interactions between prenatal and postnatal environmental influences may critically shape oxytocinergic and vasopressinergic systems, ultimately contributing to long‐term behavioural phenotypes.^41^, ^42^, ^52^, ^53^

Prenatal factors such as stress, nutritional restriction, and immune activation can alter maternal physiology and influence embryonic development through gene–environment interactions, modulate the organization and plasticity of developing systems, and play a determinant role in programming stress responsivity across the lifespan.^52^, ^54^ However, postnatal EE exposure must also be considered, as direct contact with physical, social, and sensory stimuli further shapes neurodevelopment and behaviour.^55^

Additionally, studies demonstrate that enriched dams typically exhibit enhanced maternal care, including increased licking, grooming, nursing, and the construction of more complex nests, and pup‐directed interactions, all of which have been shown to regulate offspring stress responsivity and contribute to long‐term neurodevelopment and behavioural outcomes.^17^, ^18^, ^56^, ^57^ Variations in maternal behaviours during the pre‐weaning period modulate offspring neuroendocrine systems, including the Hypothalamic–Pituitary–Adrenal axis and the OXT/AVP circuits, thereby influencing emotional behaviour and stress responsiveness later in life.^41^, ^53^, ^58^ Therefore, the behavioural and neuroanatomical changes observed in the present study may have resulted not only from the direct effects of environmental enrichment on the offspring but also from indirect effects mediated by alterations in maternal care. Because maternal behaviour was not directly quantified in the present study, its relative contribution cannot be determined. Future studies combining maternal behavioural assessment with cross‐fostering approaches will be important to disentangle the respective contributions of maternal care and direct environmental stimulation.

Finally, the multifactorial nature of perinatal environmental enrichment complicates the identification of the specific components responsible for the observed effects. Enhanced maternal care, modulation of the intrauterine environment, and direct sensory and social stimulation of the offspring likely act in concert to shape long‐term behavioural and neuroanatomical outcomes. Future studies using approaches such as cross‐fostering and selective manipulation of enrichment components will be important to disentangle the relative contribution of these mechanisms.

An additional consideration concerns the interpretation of the NE condition. Although the present study was designed to compare offspring exposed or not exposed to perinatal environmental enrichment, the observed differences may reflect not only the beneficial effects of environmental enrichment but also adaptive responses arising from contrasting early‐life housing conditions. Because the experimental design did not include a third housing condition representing conventional laboratory housing with nesting material, these alternative interpretations cannot be fully distinguished. Accordingly, the present findings are best interpreted as demonstrating the long‐term consequences of contrasting early‐life environmental conditions, rather than attributing the observed behavioural and neuroanatomical differences exclusively to environmental enrichment.

An important aspect of the present experimental design is that environmental enrichment was restricted to the perinatal period, with all animals transferred to non‐enriched housing after weaning. Accordingly, the present findings reflect the long‐term consequences of environmental enrichment restricted to early life, rather than the effects of continuous environmental enrichment. Previous studies have shown that the removal of environmental enrichment may partially attenuate behavioural and neurobiological benefits or induce adaptive changes associated with enrichment withdrawal.^59^, ^60^ Nevertheless, evidence also indicates that the beneficial effects of early‐life environmental enrichment on anxiety‐like behaviour can persist long after enrichment has ceased.^61^, ^62^ Consistent with these observations, our findings demonstrate that hypothalamic structural plasticity persisted into adulthood despite identical post‐weaning housing conditions, suggesting that transient early‐life environmental stimulation is sufficient to induce enduring developmental programming of the OXT and AVP systems. Importantly, because all experimental groups were maintained under identical non‐enriched housing conditions after weaning, the differences observed in adulthood are consistent with long‐lasting effects of the early‐life environmental manipulation rather than differences in the post‐weaning housing environment. Future studies directly comparing continuous and transient enrichment protocols will be important to distinguish the specific contributions of environmental enrichment itself from those associated with enrichment withdrawal.

Finally, an additional limitation of the present study is that potential litter effects were not explicitly incorporated into the statistical analyses. Although litters were standardized after birth and only one offspring per litter was included in the morphometric analyses, behavioural analyses were conducted at the individual‐animal level. Because littermates share common prenatal and early postnatal environments, some degree of non‐independence cannot be completely excluded. Future studies using litter as the experimental unit or mixed‐effects statistical approaches will be important to confirm the robustness of these findings.

## 5 CONCLUSIONS

Overall, these findings demonstrate that perinatal EE induces enduring neuroplastic modifications in hypothalamic OXT and AVP systems and exerts sex‐dependent effects on emotional regulation. Such plasticity may represent a modulating mechanism through which early environmental conditions shape behavioural trajectories and resilience across the lifespan.

## AUTHOR CONTRIBUTIONS

**Samantha Costa Amorim Muniz:** Conceptualization; investigation; writing – original draft; writing – review and editing; methodology; data curation; formal analysis; visualization. **João Victor Silva Nani:** Data curation; formal analysis; writing – review and editing. **Naima Jamile dos Santos Marciano:** Validation; methodology. **Raquel do Nascimento de Souza:** Investigation. **Roberto Laureano‐Melo:** Investigation. **Wellington da Silva Cortes:** Methodology; writing – review and editing. **Fábio Fagundes da Rocha:** Conceptualization; resources; funding acquisition; writing – review and editing; supervision. **André Souza Mecawi:** Conceptualization; supervision; resources; writing – review and editing; funding acquisition.

## CONFLICT OF INTEREST STATEMENT

The authors declare no conflicts of interest.

## ETHICS STATEMENT

All animal procedures were reviewed and approved by the Animal Ethics Committee of the Federal Rural University of Rio de Janeiro (CEUA‐ICBS; Protocol No. 08/2022). All experimental procedures were conducted in accordance with the institutional guidelines and the Brazilian National Council for the Control of Animal Experimentation (CONCEA) regulations for the care and use of laboratory animals.

## Supporting information


**Table S1.** Litter size at birth, litter standardization, and post‐weaning cage allocation.
**Table S2.** Summary distribution of cage size after regrouping.
**Figure S1.** Comparison of litter size at birth between the NE and PEE groups. Litter size at birth in the non‐enriched (NE) and perinatal enriched environment (PEE) groups. Each point represents one litter. Horizontal bars indicate the median. Groups were compared using a two‐tailed Mann–Whitney test. No significant difference was observed between groups (*p* = .154).

## Data Availability

The data that support the findings of this study are available from the corresponding author upon reasonable request.
