# Supplementary material for: Early‐life environmental enrichment reduces anxiety and induces sex‐dependent neuroplasticity in hypothalamic oxytocin and vasopressin neurons
Source: J Neuroendocrinol. 2026 Sep 9;38(9):e70263. doi: 10.1111/jne.70263 (PMC13557847; doi:10.1111/jne.70263)
Supplement: Supplementary file 1 [file JNE-38-e70263-s001.docx]

**Early-life environmental enrichment reduces anxiety and induces sex-dependent neuroplasticity in hypothalamic oxytocin and vasopressin neurons**

Authors:

Samantha Costa Amorim Muniz^1,3*^

João Victor Silva Nani^2^

Naima Jamile dos Santos Marciano^2^

Raquel do Nascimento de Souza^1^

Roberto Laureano-Melo^1,3^

Wellington da Silva Cortes^1,3^

Fábio Fagundes da Rocha^1,3#^

André Souza Mecawi^2#^

*Corresponding author:

Drª. Samantha Costa Amorim Muniz

Department of Biophysics, Escola Paulista de Medicina, Universidade Federal de São Paulo. 581 Botucatu, Vila Clementino, São Paulo, SP, 04023062, Brazil.

Email: [samanthacamuniz@gmail.com](mailto:samanthacamuniz@gmail.com)

ORCID: 0009-0003-1616-5731

**Supplementary Table S1.** Litter size at birth, litter standardization, and post-weaning cage allocation.

*There was no significant difference in litter size at birth between the NE and PEE groups (two-tailed Mann–Whitney test, exact *p* = **0.154**).

**Final cage composition refers to the housing arrangement of animals included in the adult behavioural and neuroanatomical experiments.

**Supplementary Table S2.** Summary distribution of cage size after regrouping

**Supplementary Figure S1.** Comparison of litter size at birth between the NE and PEE groups

Supplementary Figure S1. Litter size at birth in the non-enriched (NE) and perinatal enriched environment (PEE) groups. Each point represents one litter. Horizontal bars indicate the median. Groups were compared using a two-tailed Mann–Whitney test. No significant difference was observed between groups (p = 0.154).
